# Image-Guided Hyaluronic Acid Injection and Knee Bracing Significantly Improve Clinical Outcomes for High-Grade Osteoarthritis

**DOI:** 10.1186/s40798-015-0029-5

**Published:** 2015-09-15

**Authors:** Terry K. Morgan, Emilie Jensen, Jeong Lim, Russell Riggs

**Affiliations:** 1 Department of Pathology, Oregon Health & Science University, 3181 SW Sam Jackson, Mail Code L-471, Portland, OR 97239 USA; 2 Department of Public Health & Preventative Medicine, Oregon Health & Science University, Portland, OR USA; 3 Reflex Clinic, Portland, OR USA

**Keywords:** Knee osteoarthritis, Hyaluronic acid, Intra-articular injection, Fluoroscopy

## Abstract

**Background:**

Intra-articular hyaluronic acid (HA) injection is an intermediate option between analgesics and knee joint replacement in patients with osteoarthritis (OA). Our objective was to test whether image-guided HA injections may improve knee OA outcomes after 6 months of treatment independent of potential covariates.

**Methods:**

This is a retrospective case series with multivariate outcome-based analysis of 207 consecutive adult patients with mild to severe knee OA treated at a single out-patient clinic employing fluoroscopy-guided HA injections. We employed a customized pain (scored 0–10) and function (scored 0–120) questionnaire based on the Likert scale to compare baseline scores with 6-month outcomes. Linear and logistic (based on >9-point score improvement) regression analysis was used to adjust for potential covariates, including grade of disease, patient age, gender, body mass index, smoking history, medical history (e.g., diabetes or heart disease), use of daily pain medications, fish oil supplementation, knee bracing, and physical therapy.

**Results:**

Significant covariates included OA grade, knee bracing, and analgesic use. Most of the study subjects were women (124/207, 60 %) and obese (113/207, 55 %). Clinically significant improvements in index scores (>9 points) at 6 months were observed in more than 50 % of cases post-image-guided HA injection. Regression analysis revealed a complimentary affect with knee bracing, especially in severe grade 4 disease (odds ratio 5.5 [1.14–27.0], *P* < 0.05). Daily analgesic use reflected a poor clinical response to treatment.

**Conclusions:**

Our data suggest image-guided HA injections coupled with knee bracing may benefit patients with moderate to severe knee osteoarthritis.

**Key Points:**

- Image-guided hyaluronic acid injections significantly improve clinical outcomes at 6 months for mild, moderate, and severe knee osteoarthritis.
- Knee bracing is a significant covariate for clinical improvement in severe grade 4 disease.
- Daily analgesic use is associated with high-grade disease and less clinical improvement.

## Background

Osteoarthritis (OA) of the knee is a leading cause of adult disability [1], affecting approximately 10–15 % of Americans over the age of 60 years [2]. The estimated cost to the United States economy is over $100 billion per year [3, 4], and the prevalence of the disease is expected to increase over the next decade due to obesity and aging of the population.

A wide variety of treatment options are available [5], including weight loss, knee bracing, physical therapy, analgesics, intra-articular injections (e.g., hyaluronic acid [HA], methylprednisolone), and surgery (e.g., debridement or joint replacement). Non-surgical conservative approaches such as HA injection provide an intermediate approach between analgesics and joint replacement with the objective of decreasing pain, improving function, and delaying the need for joint replacement. Recent meta-analyses provide conflicting opinions about the efficacy of HA injections [6–8], which may affect providers’ willingness to recommend this type of treatment. However, we suspect many patients may potentially benefit from image-guided injections [9, 10] after controlling for covariates.

We hypothesized that image-guided intra-articular HA injections improve 6-month clinical outcomes in adults with mild to severe knee OA. Moreover, supplemental knee bracing and analgesic support may improve outcomes compared with image-guided HA injection alone.

## Methods

### Study Design

This is a retrospective case series with multivariate outcome-based analysis using an Oregon Health & Science University Institutional Review Board approved protocol to extract clinical chart data from 207 adult patients (25–105 years old) with mild (grade 2), moderate (grade 3), or severe (grade 4) knee OA treated at a single out-patient clinic in 2013. All study subjects were Caucasian with 6 months of clinical follow-up after a series of three fluoroscopy-guided (1.0 ml of IsoVue contrast injected into the knee with a 21-gauge needle) HA (*Euflexxa*, 1 % Sodium Hyaluronate, Ferring Pharm. Inc. Parsippany, NJ) injections administered 1 week apart by an experienced physician (RR). Clinical data were extracted from uniform patient questionnaires using a customized pain (scored 0–10) and function (scored 0–120) assessment based on the Likert scale [7, 11]. Physical examination and clinical history were screened for potential covariates, including the grade of osteoarthritis, which was defined by the Kellgren-Lawrence criteria [12]: grade 2 (mild) had definite joint space narrowing and osteophytic lipping, grade 3 (moderate) also showed sclerosis and possible bone contour deformity, and grade 4 (severe) had severe sclerosis, joint narrowing, large osteophytes, and bone contour deformities. Patient age, gender, body mass index, smoking history, significant medical history (e.g., diabetes or heart disease), subjective reporting of over-the-counter daily analgesics (e.g., NSAIDs), daily fish oil supplements, and duration of disease were also documented. More than half of the study subjects (110/207) were fitted with a non-customized single-hinged medial off-loading knee brace (V/Q OrthoCare, Vista, CA) after HA injection for the duration of the 6-month interval available for outcome metrics. Knee bracing was recommended to patients with limitations in activity defined by the ability to ambulate up and down a flight of stairs ±knee brace. Physical therapy was recommended for all study subjects. Of these, 100 study subjects regularly participated in knee-centric physical therapy (8–10 sessions over 6-month study period). Patients completed follow-up questionnaires and had a physical examination at 2 and 6 months post-image-guided HA injection. This archived patient chart data was extracted into a coded file without patient identifiers (e.g., name, birthdate) for statistical analysis. Extraction quality assurance was verified by 10 % independent review (20/207 cases).

### Statistical Analyses

All statistical analyses were performed using SAS (version 9.4, SAS Institute, Cary, NC). Patient demographics, potential covariates, and outcome metrics were examined by univariate analysis using the Student *t*-test. Potential covariates (e.g., knee bracing, analgesics) were analyzed as binary variables (yes or no). In the cases with bilateral knee involvement, the highest OA grade knee was employed for analysis. The primary outcome metric was pain and function outcomes at 2 and 6 months compared with baseline (before first HA injection) for linear regression analysis, and “clinically significant” improvement was defined as >9-point decrease in score for logistic regression analysis. Subjective pain index (0–10) was a secondary outcome analyzed by linear regression.

## Results

Multivariate regression analysis demonstrated that knee osteoarthritis grade was the most significant covariate affecting clinical outcomes at 2 months (*P* < 0.0001) and 6 months (*P* < 0.0001) post-image-guided HA injection. Therefore, all analyses were reported separately for each OA grade: mild (grade 2), moderate (grade 3), and severe (grade 4) disease (Table 1). As expected, univariate analysis showed that increasing severity of knee OA was related to increasing subject age and body mass index. Most of the study subjects were obese with BMIs ≥30.0 (113/207, 55 %) and most were women (124/207, 60 %). Univariate analysis did not show a relationship between OA grade and smoking, daily analgesic use, or physical therapy. Moderate to severe OA were more likely to be fitted with a knee brace. Surprisingly, the greatest frequency of improvement was seen in more severe disease (68 % of study subjects), which also showed the most improvement in pain index (40 % compared with 21 % in mild disease).

**Table 1:** Patient demographics and outcomes treated by image-guided hyaluronic acid injection

	OA grade 2 (*n* = 58)	OA grade 3 (*n* = 98)	OA grade 4 (*n* = 51)	*P* value *X* ^2^ or ANOVA
Age (mean, SD)	56.3 ± 12.4	64.0 ± 11.3	69.8 ± 12.2	*P* < 0.0001
Male gender (*n*, [%])	20 (35 %)	41 (42 %)	21 (42 %)	*P* = 0.62
Body mass index (mean, SD)	29.6 ± 6.5	31.2 ± 6.5	34.6 ± 9.3	*P* < 0.01
Never smoked (*n*, [%])	49 (85 %)	86 (88 %)	42 (82 %)	*P* = 0.29
Physical therapy (*n*, [%])	27 (47 %)	44 (45 %)	20 (39 %)	*P* = 0.72
Daily analgesics (*n*, [%])	23 (40 %)	41 (42 %)	26 (51 %)	*P* = 0.39
Knee bracing (*n*, [%])	20 (35 %)	60 (61 %)	31 (61 %)	*P* < 0.01
Fish oil (*n*, [%])	43 (74 %)	64 (65 %)	43 (84 %)	*P* = 0.05
Function index baseline (mean, SD)	34.7 ± 16.1	46.0 ± 15.4	48.1 ± 13.3	*P* < 0.0001
Index at 6 months (mean, SD)	27.2 ± 15.9	26.3 ± 14.9	36.4 ± 17.7	*P* < 0.01
Mean improvement (±SD)	6.7 ± 19.0	19.3 ± 16.8	12.5 ± 14.6	*P* < 0.001
Percentage improvement (±SD)	19 % ± 32 %	40 % ± 32 %	26 % ± 31 %	*P* < 0.001
>9-point improvement (*n*, [%])	32 (55 %)	67 (68 %)	26 (51 %)	*P* = 0.07
Average pain baseline (mean, SD)	5.5 ± 2.5	6.2 ± 2.1	7.1 ± 2.1	*P* < 0.01
Pain at 6 months (mean, SD)	3.9 ± 2.0	3.8 ± 2.1	5.2 ± 2.5	*P* < 0.01
Mean improvement (mean, SD)	1.66 ± 2.1	2.74 ± 2.5	2.3 ± 2.8	*P* = 0.05
Percentage improvement (±SD)	21 % ± 38 %	40 % ± 37 %	25 % ± 47 %	*P* = 0.04

Univariate analysis of patient demographics and treatment outcomes relative to knee osteoarthritis (OA) severity (grades 2–4). Data are presented as the mean ± standard deviation (SD) or percent (%) within OA grade

Regression analysis revealed that knee bracing provided a positive complimentary effect to HA injection in severe OA cases (Table 2) to improve outcomes with an odds ratio of 5.5 (1.14–27.0) and *P* value <0.05. Analgesic use reflected a poor clinical response to treatment, rather than positive additive effect as first postulated. Physical therapy significantly decreased subjective pain index for mild OA (*P* < 0.01), but it had no effect on moderate or severe disease. Outcomes at 2 months were not significantly different than 6-month outcomes in this case series (data not shown) with the exception that bracing appeared to positively affect outcomes in moderate cases at 2 months (odds ratio 2.17 [0.86–5.48], *P* value 0.02), which remained a positive effect at 6 months, but the effect was not statistically significant (Table 2). Mild OA did not respond to knee bracing at either 2 or 6 months of evaluation, although logistic regression suggested a positive trend (*P* = 0.19).

**Table 2:** Regression analysis of significant covariates for clinical improvement

	OA grade 2 (*n* = 58)		OA grade 3 (*n* = 98)		OA grade 4 (*n* = 51)	
Function index linear regression	Parameter estimate	*P* value	Parameter estimate	*P* value	Parameter estimate	*P* value
Knee bracing	+2.69	0.67	+2.56	0.51	+10.81	0.04
Daily analgesics	−5.58	0.38	−8.6	0.03	−13.94	0.003
Logistic regression (>9-point improvement)	Odds ratio [95 % C.I.]	*P* value	Odds ratio [95 % C.I.]	*P* value	Odds ratio [95 % C.I.]	*P* value
Knee bracing	2.4 [0.66–8.81]	0.19	1.22 [0.45–2.22]	0.70	5.54 [1.14–27.0]	0.03
Daily analgesics	0.12 [0.03–0.53]	0.005	0.55 [0.18–1.63]	0.28	0.12 [0.03–0.61]	0.009
Average pain index linear regression	Parameter estimate	*P* value	Parameter estimate	*P* value	Parameter estimate	*P* value
Knee bracing	+0.94	0.25	+0.30	0.67	+1.81	0.10
Daily analgesics	−0.59	0.46	−1.06	0.14	−2.15	0.03
Physical therapy	+2.11	0.004	−0.25	0.73	−1.30	0.22

In addition to intra-articular HA injection, knee bracing significantly improved clinical outcomes with positive (+) effect on clinical indices at 6 months in severe (grade 4) osteoarthritis (OA) analyzed by both linear and logistic regression. In contrast, daily analgesic use was a sign of poor clinical response to treatment with worsening scores (−)

## Discussion

Intra-articular HA knee injections are commonly performed by orthopedic surgeons [13] and increasingly by primary care physicians [5]. Although recent meta-analyses provide conflicting opinions about the efficacy of HA injections [6–8], it should be no surprise that image-guided injections may yield significantly better results [9, 10]. Our retrospective analysis of fluoroscopy-guided HA knee injections supports this conclusion. Moreover, our multivariate regression analysis reveals OA grade and bracing may significantly affect clinical outcomes.

Most of the image-guidance literature centers on corticosteroid injections. For example, Jones et al. [10] reported that ultrasound guidance yielded clinically significant improvement in 28/54 (52 %) of their study subjects, while only 7/30 (23 %) showed improvement without imaging. They attributed this discrepancy to incorrectly placed injections, which missed the joint space. Indeed, meta-analyses have shown that image-guided intra-articular knee injections yield ~97 % accuracy for needle placement compared with ~81 % accuracy without imaging [9], which of course means better efficacy and increased cost-effectiveness [14, 15].

An important limitation of our retrospective case series is the fact that all the study subjects received image-guided injections. They were not randomized into guided and non-guided HA injections for comparison. However, our data suggest future studies should test whether guidance together with knee bracing improves short-term and long-term outcomes when treating knee OA with HA. This is especially important for severe disease, which is a group most likely to require joint replacement and may benefit most from combined HA injection and knee bracing.

Analgesic use showed no positive effects in our data, and the literature suggests they have little clinical effect [16]. Notably, daily analgesic use was not prescribed in our study. Patients who reported daily analgesic use were more likely to have high-grade disease, less likely to show a functional improvement by 6 months, and less likely to report a reduction in the subjective pain index relative to baseline. We therefore suspect these patients may be more likely to be long-term non-responders to HA injection and knee bracing. This data also suggest the mechanism of action of HA injections may not be solely anti-inflammatory.

The biological actions of HA viscosupplementation are only beginning to be understood. For example, HA may increase synovial fluid viscosity [17] and regulate a number of cytokines involved in both anti-inflammatory activity and extracellular matrix restorative mechanisms. Recent studies exploring the effects of HA on activated CD4 positive T cells in synovial fluid [18] and reduction of joint tissue catabolism [19] are intriguing. Both mechanisms suggest that in some patients HA may have more long-term positive effects than only short-term improvements in pain and patient function. Long-term outcome data were not available for our analysis, but they are needed to test whether image-guided HA injections with knee bracing may delay joint replacement. Over 75 % of OA patients with joint space narrowing progress to total knee replacement within 18 years of diagnosis [20]. Delaying joint replacement is therefore very important because the lifespan of the device is 10–15 years. In an obese aging population, cost-effective interventions like HA injection and knee bracing make sense.

## Conclusions

Our data suggest image-guided HA injections coupled with knee bracing may benefit patients with moderate to severe knee osteoarthritis. With the current rates of obesity and the advanced aging of the population, it is reasonable to speculate that many more patients will require revision surgery if alternative intervention methods like viscosupplementation are not effective. A randomized prospective trial to thoroughly evaluate the short-term and long-term impact of image-guided HA injection with and without knee bracing is needed.

## Abbreviations

BMI: body mass index
HA: Hyaluronic acid
OA: Osteoarthritis
